# Clinical Use of Next-Generation Sequencing in the Diagnosis of Wilson's Disease

**DOI:** 10.1155/2016/4548039

**Published:** 2015-12-24

**Authors:** Dániel Németh, Kristóf Árvai, Péter Horváth, János Pál Kósa, Bálint Tobiás, Bernadett Balla, Anikó Folhoffer, Anna Krolopp, Péter András Lakatos, Ferenc Szalay

**Affiliations:** ^1^1st Department of Internal Medicine, Semmelweis University, Koranyi Sandor Street 2/a, Budapest 1083, Hungary; ^2^PentaCore Lab, Koranyi Sandor Street 2/a, Budapest 1083, Hungary

## Abstract

*Objective*. Wilson's disease is a disorder of copper metabolism which is fatal without treatment. The great number of disease-causing *ATP7B* gene mutations and the variable clinical presentation of WD may cause a real diagnostic challenge. The emergence of next-generation sequencing provides a time-saving, cost-effective method for full sequencing of the whole *ATP7B* gene compared to the traditional Sanger sequencing. This is the first report on the clinical use of NGS to examine *ATP7B* gene. *Materials and Methods*. We used Ion Torrent Personal Genome Machine in four heterozygous patients for the identification of the other mutations and also in two patients with no known mutation. One patient with acute on chronic liver failure was a candidate for acute liver transplantation. The results were validated by Sanger sequencing. *Results*. In each case, the diagnosis of Wilson's disease was confirmed by identifying the mutations in both alleles within 48 hours. One novel mutation (p.Ala1270Ile) was found beyond the eight other known ones. The rapid detection of the mutations made possible the prompt diagnosis of WD in a patient with acute liver failure. *Conclusions*. According to our results we found next-generation sequencing a very useful, reliable, time-saving, and cost-effective method for diagnosing Wilson's disease in selected cases.

## 1. Introduction

Wilson's disease (WD) is a rare autosomal recessive disorder of copper metabolism.* ATP7B* gene mutation is in the background of the excessive copper accumulation which is fatal without treatment. More than 550 disease-causing mutations of the gene located at chromosome 13q14.3-q21.1 consisting of 21 exons have been identified [1].

The geographical distribution of the mutations of the* ATP7B* gene is inhomogeneous [2–5]. In Hungary the p.His1069Gln mutation is the most frequent one with 71% prevalence among the patients [6].

The variable clinical presentation of WD may cause a real diagnostic challenge. The suspicion of the disease usually arises when hepatic or neurologic-psychiatric symptoms appear. Low ceruloplasmin level and presence of Kayser-Fleischer ring could support the diagnosis, but in many cases only genetic testing could confirm it. Genetic investigation of asymptomatic siblings has an extreme importance, since the early treatment could prevent the manifestation of the disease [7]. In acute liver failure urgent genetic testing of all known mutations may strengthen the diagnosis of Wilson's disease.

The emergence of next-generation sequencing (NGS) provides a time-saving, cost-effective method for full sequencing of the whole* ATP7B* coding sequence compared to the traditional Sanger sequencing. The NGS technology is based on the detection of a signal during the synthesis of the DNA strand, and therefore the synthesis does not need to be terminated for the perception. On the other hand, several DNA strands can be examined simultaneously [8].

This is the first report on the clinical use of NGS to examine* ATP7B* gene in WD patients including doubtful cases. We used Ion Torrent Personal Genome Machine in heterozygous patients for the identification of the other mutations and also in patients with no known mutation including one with acute on chronic liver failure.

## 2. Materials and Methods

The method we used for the genetic testing has been previously published by our group for screening of neurofibromatosis type 1 gene [9].

### 2.1. Biological Samples and DNA Isolation

Six (five male and one female) WD patients, four heterozygous for* ATP7B* p.His1069Gln mutation identified by fast PCR test and two with unknown mutation, were selected for this study. The patients were diagnosed and treated at the 1st Department of Internal Medicine, Semmelweis University, Budapest. The diagnosis was based on the international WD score system published in 2003 [10], and each patient had 4 or more scores. The study was approved by the Semmelweis University's Committee of Research Ethics and was conducted in accordance with the Helsinki Declaration. All patients gave written informed consent.

Genomic DNA was isolated from 200 *μ*L of peripheral blood using ReliaPrep Blood gDNA Miniprep System (Promega, Madison, WI). Briefly, the blood samples were digested with Proteinase K solution in the presence of Cell Lysis Buffer, and, after 10 min of incubation at 56°C, DNA was bound to ReliaPrep Binding Column. After three washes, DNA was eluted into 50 *μ*L of nuclease-free water. The concentration of the isolated DNA was determined with Qubit dsDNA HS Assay Kit (Life Technologies, Carlsbad, CA).

### 2.2. Ion Torrent Sequencing

*ATP7B* (21 coding exons) amplicons were designed using the AmpliSeq Designer software (Life Technologies, CA, USA), targeting the complete coding sequence of* ATP7B* gene, resulting in a total of 55 amplicons. To gain a higher coverage of the coding exons, we designed the primers to also flank some parts of the introns. Amplicon library was prepared using the Ion AmpliSeq Library Kit 2.0 (Life Technologies, CA, USA); briefly, multiplex primer pools were added to 10 ng of genomic DNA and amplified with the following PCR cycles: at 99°C for 2 min, at 99°C for 15 s, and at 60°C for 4 min (18 cycles), and holding on at 10°C. Primers were partially digested using a FuPa reagent, and then sequencing adapters were ligated to the amplicons. The library was purified in multiple times using the Agencourt AMPure XP Reagent (Beckmann Coulter, CA, USA). The concentration of the final library was determined by fluorescent measurement on Qubit 2.0 instrument (Life Technologies, CA, USA). Template preparation was performed with Ion OneTouch kit (Life Technologies, CA, USA) on semiautomatic Ion OneTouch instrument using an emPCR method. After breaking the emulsion, the nontemplated beads were removed from the solution during the semiautomatic enrichment process on Ion OneTouch ES (Life Technologies, CA, USA) instrument. After adding the sequencing primer and polymerase, the fully prepared Ion Sphere Particle (ISP) beads were loaded into an Ion 314 v2 sequencing chip, and the sequencing runs were performed using the Ion PGM 200 Sequencing kit v2 (Life Technologies, CA, USA) with 500 flows.

### 2.3. Sanger Sequencing Validation

The PCR primers were designed using Primer3Plus (http://primer3plus.com/) software. Roche FastStart TaqMan Probe Master (Roche) kit was used to amplify the target regions and the PCR program was as follows: 95°C for 10 min, 40 cycles of 95°C for 30 s, 60°C for 30 s, and 72°C for 45 s, and the final step was 72°C for 5 min. PCR products were enzymatically cleaned using ExoSAP IT (Affymetrix, Santa Clara, CA) according to the manufacturer's instructions. Sanger sequencing was performed using BigDye Terminator v3.1 Cycle Sequencing Kit (Life Technologies) using an ABI 3130 instrument (Life Technologies).

### 2.4. Data Analysis

Data from the Ion Torrent runs were analyzed using the platform-specific pipeline software Torrent Suite v3.6 for base calling, trim adapter and primer sequences, filter out poor quality reads, and demultiplex the reads according to the barcode sequences. Briefly, TMAP (https://github.com/iontorrent/TMAP) algorithm was used to align the reads to the hg19 human reference genome, and then the variant caller plug-in was selected to run to search for germ line variants in the targeted regions. The variant caller algorithm parameters were more relaxed to avoid false negative cases. Integrative Genomics Viewer was used for visualization of the mapped reads. Variants were reviewed and annotated using dsSNP (http://www.ncbi.nlm.nih.gov/projects/SNP/) and Wilson Disease Mutation Database (http://www.wilsondisease.med.ualberta.ca/index.asp). For variant interpretation, Ingenuity Variant Analysis Pipeline (Ingenuity, Rewood City, CA) was also used. Pathogenic status of the variant was stated if it was a missense variant with <1% minor allele frequency and/or the variant was listed in the literature or in the databases as a pathogenic alteration. All of the deleterious variants were confirmed by Sanger sequencing. The Sanger sequence data were investigated using ABI Sequence Scanner 1.0 (Life Technologies) and BioEdit (http://www.mbio.ncsu.edu/bioedit/bioedit.html) software.

## 3. Results

The demographic and clinical characteristics of the patients are shown in Table 1. One patient without known mutation was critically ill with acute on chronic liver disease. The typical laboratory findings (ALT 90, AST 178, ALP 88, and bilirubin 247) proposed Wilson's disease. The diagnosis was strengthened by genetic testing making possible the liver transplantation via Eurotransplant program in* Patient 5* with acute on chronic liver failure.

In each case, the diagnosis of Wilson's disease was confirmed by identifying the mutations in both alleles. The results were available within 48 hours.

The average read number per sample was 134386, with an average 1X on-target coverage of 99.46%. The mean raw accuracy was 99.2%. The average base coverage depth was 1883 (Table 2). The number of identified variants per sample was between 8 and 13; however most of them were known as non-disease-causing variants.

**Figure 1:**
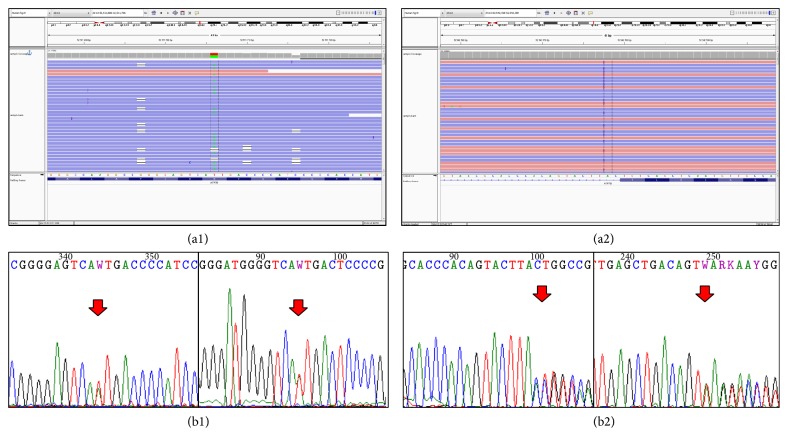
The identified mutations of patient 5. Both c.3809A>T (causing amino acid change p.Ala1270Ile) and c.1707+2dupT mutations are confirmed by Sanger-sequencing. The 3809A>C and A>G mutations are known, but the A>T substitution is a novel alteration at this position. (a1) Visualizing the alignment of the sequencing reads covering the* ATP7B* c.3809A>T heterozygous point mutation. The coverage was 400-fold (211-fold reference and 189-fold variant coverage). (b1) Validating our finding with Sanger sequencing, red arrow indicates the position of the point mutation. The mutation is present in both directions. (a2) Visualizing the alignment of the sequencing reads covering the* ATP7B* c.1707+2dupT heterozygous insertion mutation. The coverage was 399-fold (188-fold reference and 211-fold variant coverage). (b2) Validating our finding with Sanger-sequencing, red arrow indicates the position of the insertion. The mutation is present in both directions.

Overall, we found nine disease-causing variants. The most frequent mutation was p.His1069Gln (exon 14, ATP loop) detected in four patients. One novel missense mutation (p.Ala1270Ile, exon 18, ATP hinge vide Figure 1) and three well-known missense mutations (p.Arg969Gln, exon 13, TM6; p.Ala1063Val, exon 14, ATP loop, and p.Leu1305Pro, exon 19 bet ATP hinge/TM7), three frame-shift mutations (c1707+2dupT, exon 4, Cu6; p.Met769-fs exon 8 TM4, and p.Ala1135-fs exon 15 ATP loop), and one nonsense mutation (p.Gln1351Stop, exon 20, TM8) were detected. All of these variants had been validated by Sanger sequencing.

## 4. Discussion

Although there is an international diagnostic score system for WD [10], the set-up of the diagnosis remains a great challenge in many cases. The signs and symptoms are very colorful, and most of the criteria have relatively low sensitivity and/or specificity. Although genetic testing in itself can ascertain the diagnosis, it is limited by the great variety of the mutations. It is also difficult to screen the siblings of a WD patient, especially of those who do not have identified mutations, since the abnormal laboratory results of copper metabolism may occur in heterozygous carriers. The tight observation of these siblings and the doubt if they are affected can make their life very stressful and uncomfortable. The detection of the mutations in the index patient and searching for the same in the siblings can resolve this problem.

The whole gene analysis of* ATP7B* by PCR and capillary sequencing in a large cohort of WD patients has been recently published [11]. According to our knowledge based on PubMed data this is the first report on next-generation sequencing of the* ATP7B* gene for genetic diagnosis of Wilson's disease in a clinical setting. Since the disease-causing mutations may occur in the whole length of the gene and every exon could be affected, the genetic examination by classical methods is ponderous and time-consuming.

Our study clearly shows the great benefit of NGS. The compound heterozygosity has been proved in each patient within a very short examination time. Previously, we published that p.His1069Gln mutation is most common one in Hungary (71%) similar to other Central and Eastern European countries [6, 12–14]. Results of this study are in concordance with the former epidemiological data, since this mutation was confirmed in the majority of the cases, in 4 out of 6. Among the eight other mutations we found, there is one novel mutation in exon 18 which is a missense mutation causing an asparagine-isoleucine change in the transporter. Interestingly, the mutations beyond p.His1069Gln occurred only in one allele of a single patient.

However, it is already well known that p.His1069Gln homozygous mutation tends to relate with neurological symptoms; the effect of other infrequent mutations on the phenotype is hard to be examined due to the low number of cases (vide Table 1) [15].

p.Ala1063Val mutation detected in one patient who has been diagnosed with WD prior to genetic testing is thought to be a non-disease-causing variant according to the Wilson Disease Mutation Database, although only one publication suggested that it might be a polymorphism [4]. On the other hand, subsequent data show that it might be a variant of unknown significance (VUS) [16]. Furthermore it was the one and only nucleotide change in a WD family analyzed by Loudianos et al. [17]. Overall it seems that p.Ala1063Val mutation still might be associated with Wilson's disease.

NGS gave a tremendous benefit for a 47-year-old patient with acute on chronic liver failure. Although nearly all patients with ALF due to Wilson's disease are potentially diagnosed (or suspicion is very high) with use of simple biochemical and laboratory criteria (ratio of alkaline phosphatase to bilirubin, ratio of AST to ALT, and Coombs negative hemolytic anemia) [18], the diagnosis may require an urgent genetic testing of all mutations. In some patients the laboratory data alone cannot give enough scores in the international score system [10], which is required by Eurotransplant program for donor liver allocation. In our case, the results of D-penicillamine test and the NGS arrived simultaneously, confirming Wilson's disease. According to the actual regulation of Eurotransplant Organization in case of acute on chronic liver failure only WD and Budd-Chiari syndrome are accepted as indication for urgent transplantation. Identifying mutations in both alleles gave a clear-cut evidence of the disease despite lack of Kayser-Fleischer ring, lack of neurological symptoms, and p.His1069Gln mutation. Thanks to the quick diagnosis the patient has been transplanted within two days and survived, and he is still in good condition one year later.

## 5. Conclusion

According to our results we found next-generation sequencing to be a very useful, reliable, time-saving, and cost effective method for diagnosing Wilson's disease in selected cases.

## Figures and Tables

**Table 1:** Demographic and clinical characteristics of the patients.

	Gender	Age at onset (year)	KFR	Neu	HA	Urin Cu	Biopsy	Cerul (g/L)	*ATP7B* status	WD score^a^	Phenotype
Patient 1	Female	12	P	A	A	++	ND	0.18	p.Met769-fs/p.His1069Gln	6	S
Patient 2	Male	17	A	P	A	+	ND	0.05	p.Ala1063Val/p.His1069Gln	6	N1
Patient 3	Male	8	P	A	A	++	+^b^	0.06	p.His1069Gln/p.Gln1351Stop	8	H2
Patient 4	Male	17	P	A	A	+	ND	0.03	p.Ala1135-fs/p.Leu1305Pro	5	H2
Patient 5	Male	44	A	A	A	++	ND	0.08	p.Ala1270Ile/c.1707+2dupT	4	H1
Patient 6	Male	14	P	P	A	ND	ND	0.04	p.Arg969Gln/p.His1069Gln	7	N2

KFR: Kayser-Fleischer ring; Neu: neurological signs and/or CT/MRI alterations; HA: hemolytic anemia; Urin Cu: urinary copper, 1-2X ULN: +, >2x ULN or positive D-penicillamine challenge: ++; Cerul: ceruloplasmin, P: present; A: absent; ND: not done; S: sibling; H1: acute liver failure; H2: chronic liver disease; N1: neurological symptoms with liver disease; N2: only neurological symptoms.

^a^According to the international score system, 4 or more scores, diagnosis of WD is highly likely. ^b^Rhodanine positivity.

**Table 2:** Per sample and per amplicon coverage data.

Chromosome	Amplicon start	Amplicon end	Amplicon ID	Gene ID	Patient 1	Patient 2	Patient 3	Patient 4	Patient 5	Patient 6
chr13	52520358	52520440	AMPL1102520485	ATP7B	812	484	378	9467	3557	3275
chr13	52542530	52542653	AMPL1102672008	ATP7B	951	390	145	3149	5510	3183
chr13	52538909	52539030	AMPL1404436522	ATP7B	9	2	2	2	4	46
chr13	52523735	52523838	AMPL561308261	ATP7B	417	285	89	245	683	2611
chr13	52518244	52518356	AMPL561308388	ATP7B	1140	520	339	7934	6166	3595
chr13	52516492	52516613	AMPL561312436	ATP7B	600	309	70	1510	3218	2152
chr13	52515134	52515253	AMPL561312709	ATP7B	799	287	74	1340	3258	2733
chr13	52520435	52520563	AMPL561312859	ATP7B	622	308	149	409	906	2130
chr13	52511616	52511742	AMPL561313522	ATP7B	343	233	58	1462	2168	1069
chr13	52548475	52548562	AMPL561315457	ATP7B	740	557	248	660	1251	3869
chr13	52548014	52548135	AMPL561316164	ATP7B	506	109	93	244	614	3077
chr13	52535952	52536073	AMPL561319098	ATP7B	345	332	62	1845	3919	1350
chr13	52532445	52532575	AMPL561319439	ATP7B	495	382	75	260	477	2421
chr13	52518332	52518433	AMPL561320185	ATP7B	445	574	270	852	1305	2147
chr13	52511401	52511536	AMPL561321003	ATP7B	899	422	181	3036	4216	2756
chr13	52523839	52523934	AMPL561322321	ATP7B	802	389	181	3872	3781	2867
chr13	52520556	52520638	AMPL561322791	ATP7B	836	630	362	9187	4465	3540
chr13	52534277	52534394	AMPL561324803	ATP7B	670	295	126	3402	3937	2458
chr13	52542654	52542747	AMPL561326888	ATP7B	755	503	272	811	1791	3775
chr13	52524407	52524535	AMPL561327019	ATP7B	215	139	38	126	250	1023
chr13	52516614	52516708	AMPL561328057	ATP7B	546	401	206	591	1259	2779
chr13	52515254	52515365	AMPL561328062	ATP7B	768	466	191	508	1239	3144
chr13	52513223	52513345	AMPL561328524	ATP7B	454	382	113	239	732	1771
chr13	52548563	52548672	AMPL561329883	ATP7B	836	384	149	3334	4618	3056
chr13	52511743	52511824	AMPL561330934	ATP7B	718	556	323	946	1461	3040
chr13	52532576	52532683	AMPL561335846	ATP7B	671	433	227	5882	4370	2749
chr13	52511497	52511615	AMPL561335849	ATP7B	813	494	353	857	1761	3297
chr13	52549016	52549114	AMPL561337443	ATP7B	453	347	167	3818	3121	2050
chr13	52534395	52534476	AMPL561338245	ATP7B	734	542	476	1282	2378	2732
chr13	52539048	52539119	AMPL561339230	ATP7B	532	448	394	9393	3563	2527
chr13	52544567	52544690	AMPL561342268	ATP7B	806	449	130	2523	3519	2674
chr13	52548673	52548782	AMPL561343055	ATP7B	512	446	158	513	1052	3022
chr13	52548136	52548260	AMPL561345064	ATP7B	826	442	128	2692	4578	2624
chr13	52549115	52549227	AMPL561347059	ATP7B	469	369	144	398	771	2612
chr13	52539120	52539203	AMPL561347740	ATP7B	787	737	877	3031	2553	4243
chr13	52509711	52509847	AMPL561353128	ATP7B	329	280	70	208	669	2063
chr13	52549228	52549346	AMPL561354995	ATP7B	720	232	59	900	2717	2418
chr13	52508853	52508964	AMPL561358361	ATP7B	660	455	181	4930	3832	2325
chr13	52548783	52548894	AMPL561361353	ATP7B	1286	639	267	7815	7277	3588
chr13	52544691	52544813	AMPL561365512	ATP7B	542	354	150	336	898	2919
chr13	52508959	52509084	AMPL561366430	ATP7B	694	574	217	630	1344	2208
chr13	52524093	52524178	AMPL561367088	ATP7B	368	315	5	31	82	2778
chr13	52524179	52524298	AMPL561373391	ATP7B	725	390	108	2697	4100	2724
chr13	52544814	52544931	AMPL561373418	ATP7B	235	52	34	618	1093	1651
chr13	52548893	52549018	AMPL561375011	ATP7B	498	471	103	239	550	2166
chr13	52509084	52509181	AMPL561375394	ATP7B	719	461	144	5037	3879	2163
chr13	52531644	52531756	AMPL561379526	ATP7B	512	234	80	1601	2656	2155
chr13	52548255	52548381	AMPL561399016	ATP7B	467	446	98	286	676	2965
chr13	52548382	52548474	AMPL561401395	ATP7B	247	124	69	1000	357	2414
chr13	52513106	52513229	AMPL561308165	c.3699+27T>C, ATP7B	560	372	88	1778	3799	2176
chr13	52585387	52585514	AMPL561308108	c.-36C>T, c.-75A>C, ATP7B	294	287	110	313	616	2859
chr13	52585831	52585931	AMPL1275480480	ATP7B	493	397	129	807	780	1389
chr13	52585851	52585971	AMPL1275480698	ATP7B	458	140	55	1205	1642	811
chr13	52534093	52534223	AMPL1275484758	ATP7B	140	230	35	69	187	2149
chr13	52585478	52585613	AMPL561317674	ATP7B	560	186	58	997	1619	1979
